# Arthroplasty of a Charcot Knee in a Patient With Congenital Insensitivity to Pain

**DOI:** 10.7759/cureus.24116

**Published:** 2022-04-13

**Authors:** Mohammed s Alghamdi, Bashar Reda, Saeed N Albukhari, Mahmood A Qoqandi

**Affiliations:** 1 Orthopedic Surgery, King Abdulaziz University Hospital, Jeddah, SAU; 2 Medicine, King Abdulaziz University Hospital, Jeddah, SAU; 3 College of Medicine, King Saud Bin Abdulaziz University for Health Sciences, Jeddah, SAU; 4 Orthopedic Surgery, Alnoor Hospital, Makkah, SAU

**Keywords:** neuropathic arthropathy, congenital insensitivity to pain, hereditary sensory and autonomic neuropathies (hsan), total knee replacement, arthroplasty, charcot joint, charcot knee

## Abstract

Hereditary sensory and autonomic neuropathies (HSANs) include hereditary disorders that cause congenital insensitivity to pain. Moreover, patients diagnosed with such disorders are known to have genetic mutations that alter their deep pain sensation, making them more prone to developing bone and joint complications such as repetitive fractures, joint swelling, and Charcot arthropathy. Neuropathic arthropathy (Charcot joint) is a rare and relatively poorly understood condition; it is suggested to be caused by autonomic dysfunction and repetitive microtrauma and characterized by instability and joint destruction. Diagnosing the idiopathic Charcot joint is challenging and is considered to be a diagnosis of exclusion. In addition, there are limited cases of Charcot knees managed by arthroplasty. Patients with Charcot knees are commonly characterized by profound bone loss, diffuse synovitis, and instability in the knee joint. In this article, we report the case of a 13-year-old patient with known *NTRK1* gene mutation who presented with recurrent knee joint swelling episodes and instability without pain. She was diagnosed with Charcot knee joint and underwent right hinged total knee replacement. At one-year follow-up, she continued to have good knee stability and an overall functional gait. Our findings suggest that managing Charcot knee joint with total knee replacement in patients with HSAN may show improvement in terms of stability, swelling, and overall gait.

## Introduction

Congenital insensitivity to pain is a medical condition belonging to a group of rare hereditary sensory and autonomic neuropathies (HSANs) [1]. Patients having congenital insensitivity are characterized by genetic mutations that lead to the lack of deep pain sensation [2]. A well-known feature in HSAN is the loss of large myelinated and unmyelinated nerve fibers. In addition, such patients often suffer from bone and joint complications such as repetitive fractures and Charcot arthropathy [2]. Neuropathic arthropathy (Charcot joint) is a rare and relatively poorly understood condition; it is suggested to be caused by autonomic dysfunction and repetitive microtrauma and is characterized by instability and joint destruction [3,4]. In addition, a Charcot joint is a major dilemma for orthopedic surgeons [3,4] and is mainly caused by diabetes mellitus and neurosyphilis [5]. Idiopathic Charcot joint without a known secondary cause is extremely rare and may lead to a delay in diagnosis as well. Therefore, the few cases reported of idiopathic Charcot knee have presented with profound bone loss and diffuse synovitis [4,6]. The diagnosis of a Charcot knee is challenging due to the variable possible clinical presentations and is mainly based on a diagnosis of exclusion [4,5]. Classical neuropathic arthropathy is painless; however, Charcot knee joint patients usually complain of instability and pain, mainly during walking [3]. The general approach to diagnose a Charcot knee is to conduct a thorough clinical history and physical examination, aided by radiographic imaging that demonstrates clues to the diagnosis [3]. Knee dislocation or subluxation in addition to bony destruction or heterotopic ossification on imaging is sufficient to establish the diagnosis without the need for advanced imaging techniques [3]. Historically, the management of Charcot knees is either conservative or arthrodesis-based; arthroplasty has generally been avoided in such cases but has gained popularity in recent years [3]. To our knowledge, the number of published cases involving knee Charcot joint managed by arthroplasty is limited, which indicates the condition's rarity and the importance of reporting such cases. In this report, we present the case of a 13-year-old girl with a known *NTRK1* homozygous gene mutation who developed neuropathic osteoarthropathy and was treated with a hinged total knee replacement. An informed consent was obtained from the patient's parents.

## Case presentation

**Figure 1:**
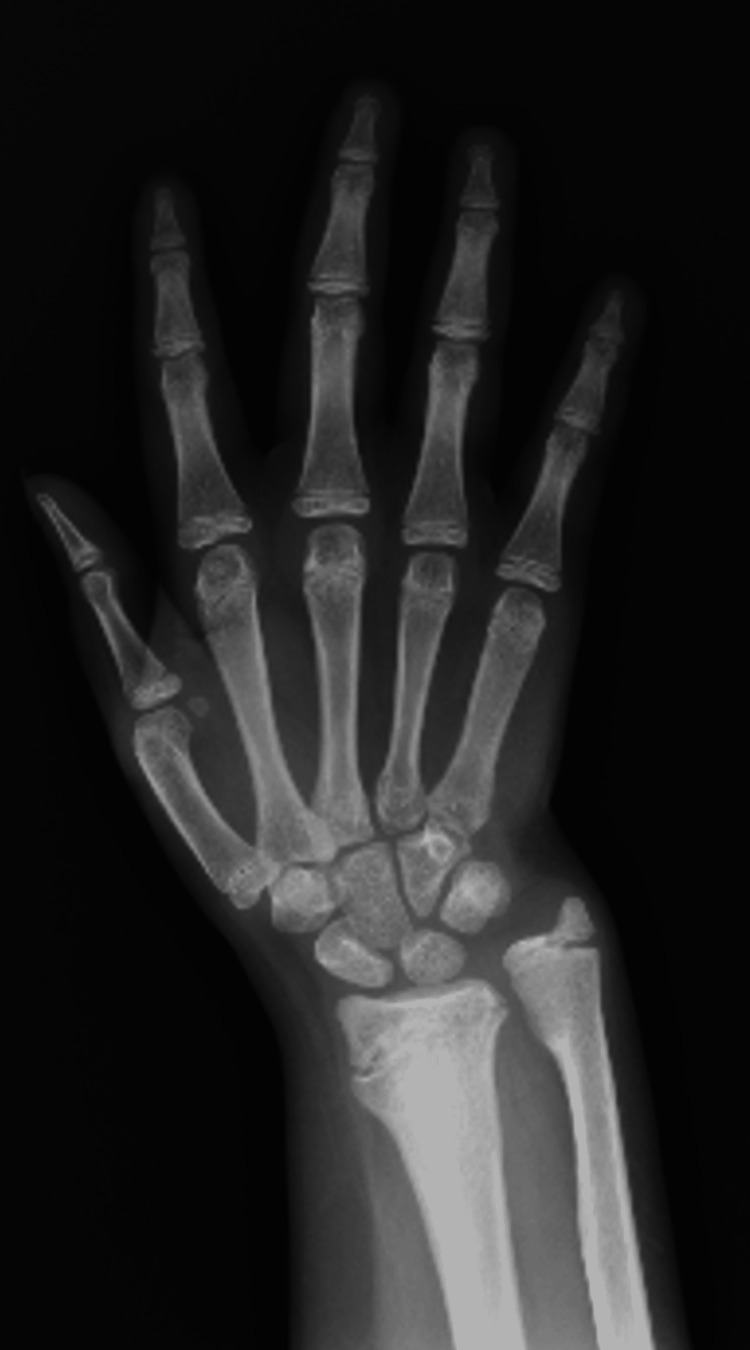
Wrist and hand X-ray for bone age.

**Figure 2:**
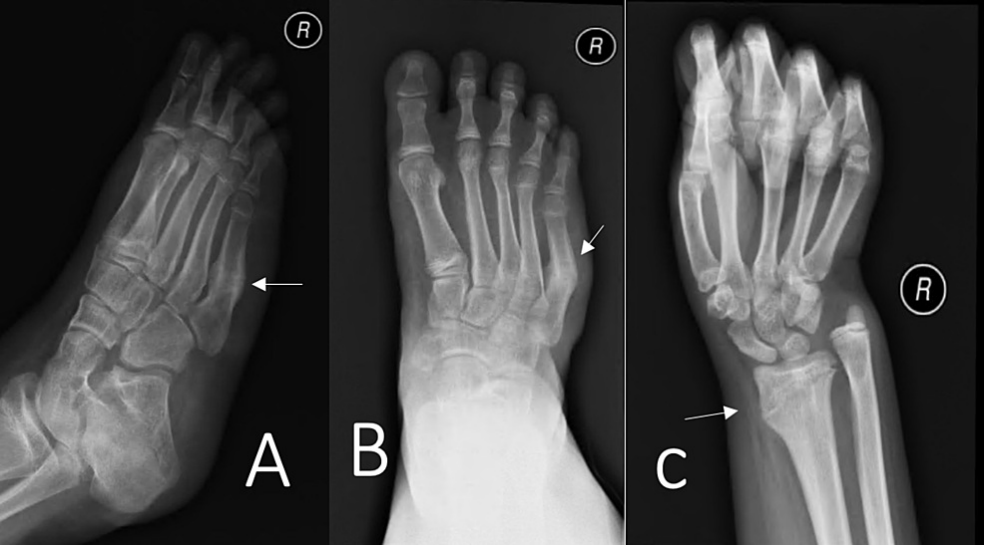
Full skeletal survey was conducted, which showed multiple fractures at different stages of healing. The patient denied any history of pain but rather incidental falls while playing.

**Figure 3:**
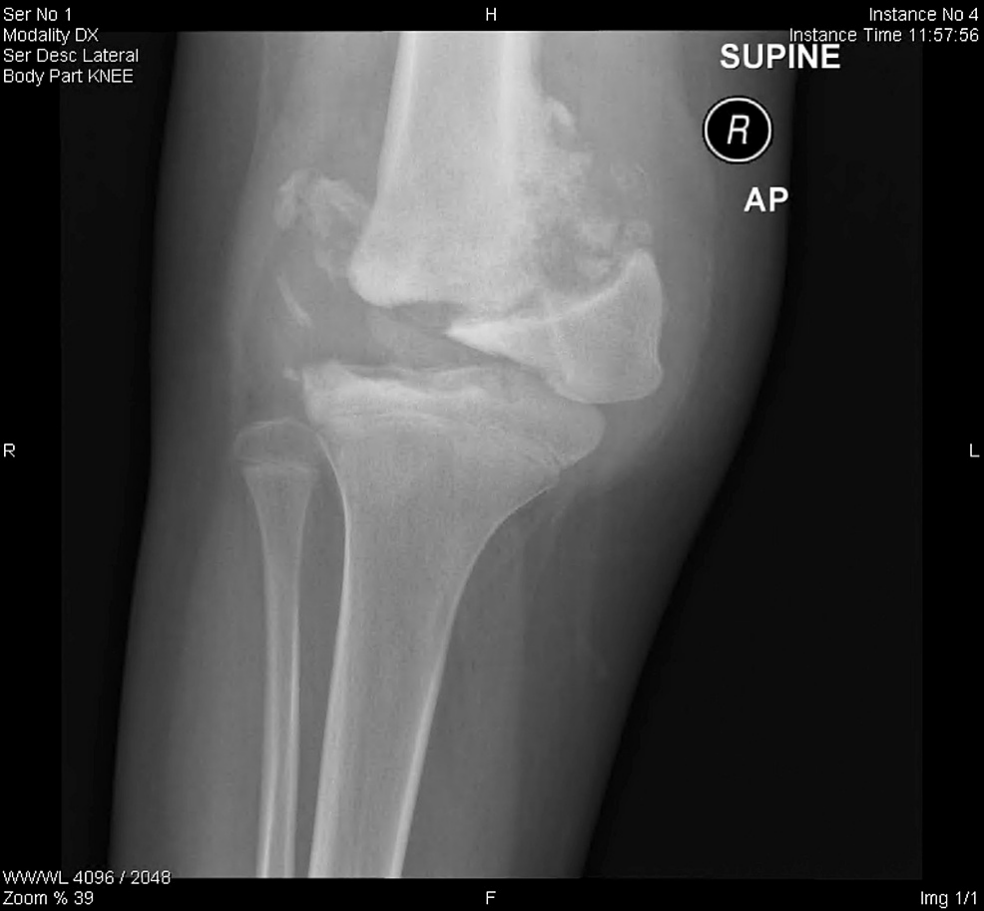
X-ray (anteroposterior view) of the right knee.

**Figure 4:**
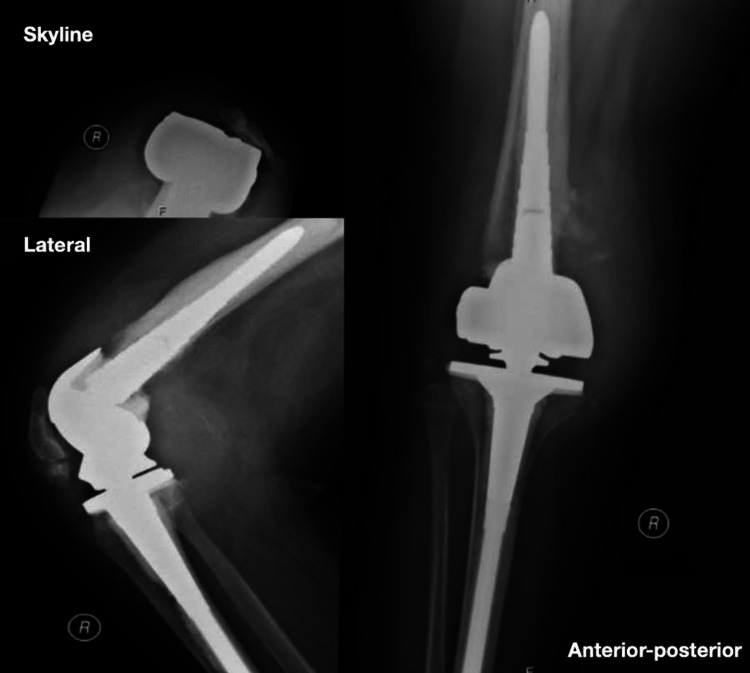
Post-operative X-rays showing the hinged implant that had been used.

**Figure 5:**
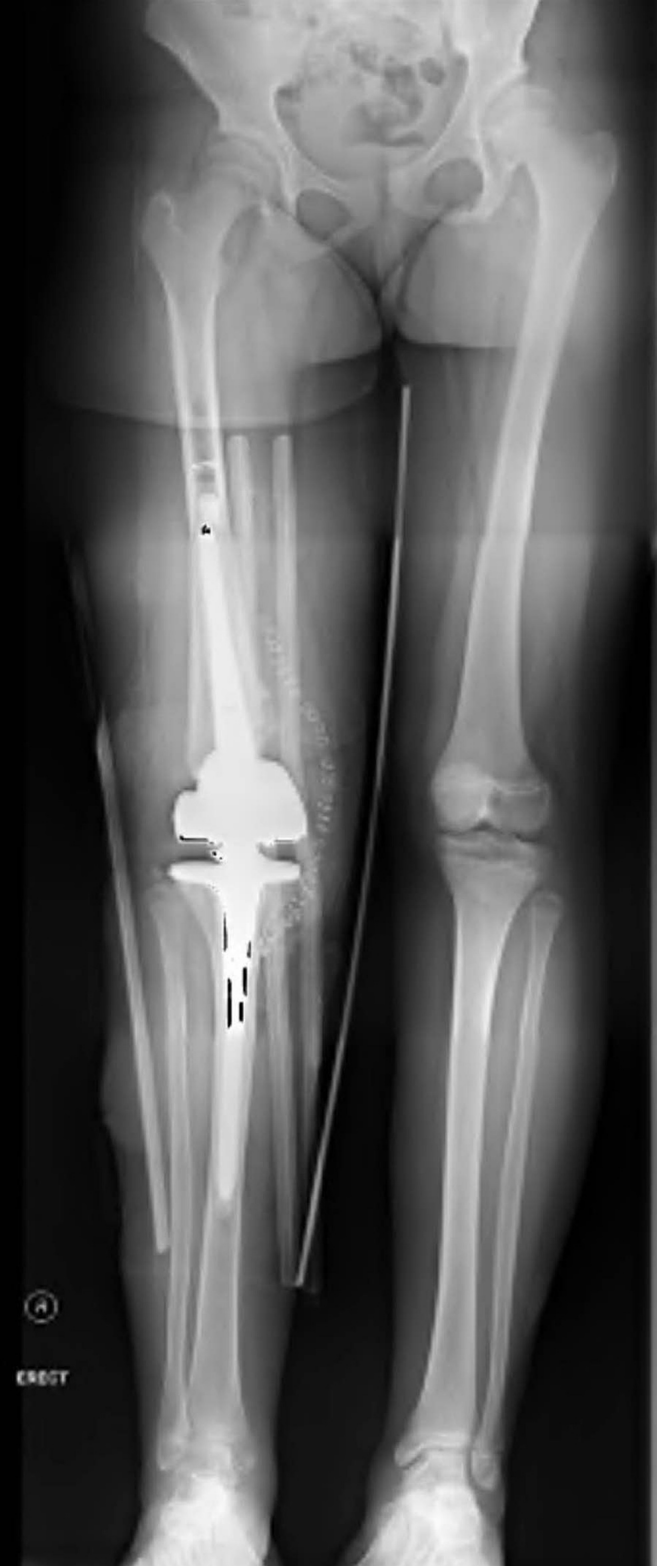
Full-length anteroposterior radiograph of both lower limbs showing restored alignment of the right knee.

A 13-year-old female presented to our clinic complaining of a limp and recurrent episodes of swelling in her right knee for the past four years. Her limp was painless, and it was associated with instability. The patient had tonsillectomy with adenoidectomy in 2013. She has hypothyroidism, which is controlled on levothyroxine 50 mcg daily. She had no allergies and took no herbal medications. She lives with her parents with two healthy siblings. Her parents are cousins with no similar history of such presentation in the family. She had no history of drug abuse or cigarette smoking. Prior to her presentation to our institution, she was diagnosed with pigmented villonodular synovitis (PVNS) after a diagnostic knee arthroscopy, which was performed outside our institution in which they performed partial synovectomy through arthroscopy. By the time she presented to our institution, she had started to develop more frequent episodes of painless joint swelling with instability. On examination, the patient had marked swelling, with no warmth or erythema. She had limitation of both passive and active range of motion, with painless multidirectional instability. Her distal neurovascular examination was intact, with strength being 5 out of 5 based on the Oxford grading scale, and all the dermatomes were intact. Full septic screen, including complete blood count, C-reactive protein, and erythrocyte sedimentation rate, and knee cultures were unremarkable and negative. Serology was found to be negative. Hand and wrist X-ray, which were performed to measure the skeletal maturity of the patient, showed that the estimated bone age was normal (Figure 1). Moreover, the patient was referred for consultation to the pain management clinic due to the detection of unreported pain associated with recurrent fracture pattern. A full skeletal survey, as shown in Figure 2, was ordered to rule out any additional unrecognized fractures. Genetic testing was ordered by the pain management clinic, and an *NTRK1* gene mutation responsible for congenital insensitivity to pain was discovered. Confirmatory histology of the PVNS was ordered with repeating knee X-ray (Figure 3). Histological report confirmed PVNS, and knee X-ray showed evidence of a Charcot joint. Moreover, a pre-operative X-ray lower limb long film was performed, which showed leg length discrepancy with 33.2 cm and 39.2 cm for right and left femurs, respectively. Our patient underwent a trial of conservative treatment with hinged braces for two years. However, frequent falls, instability, and no improvement in the gait were reported. Knee fusion or arthrodesis was avoided because it causes limitation in the range of motion, which was not desired by the patient or by her parents. Due to her young age, desire to regain full range of motion, and her family’s concern about regaining stability and mobility, hinged total knee arthroplasty was decided instead of arthrodesis. The patient underwent right hinged total knee replacement. Figures 4, 5 show the post-operative radiological images. She was followed up for one year during which she did well with good knee stability, complete passive and active range of motion, and overall gait. Moreover, the knee joint effusion also subsided.

## Discussion

HSANs are rare hereditary disorders characterized by the degeneration of unmyelinated and myelinated nerve fibers, resulting in a loss of deep sensation and pain [1,3]. HSAN was previously referred to as congenital indifference to pain, but this term was found to be misleading because the patients had congenital peripheral nerve dystrophy not an abnormally disturbed increased or decreased response to pain [2]. Due to their congenital insensitivity to pain, HSAN patients have been found to exhibit a variety of bone and joint complications [1,3]. According to Dyck et al., HSANs are classified into five subtypes based on their mode of inheritance, neuropathology, and clinical manifestations [7]. Orthopedic manifestations of HSANs are best classified under type 5, which are known to be caused by a recently discovered mutation in the nerve growth factor beta gene located in chromosome 1 [2]. Patients with HSAN type 5 are characterized by having intact body sensations except for a loss in pain and temperature sensations [1]. Inheritance of type 5 HSAN is autosomal recessive, and those patients appear to have normal muscle strength, nerve conduction studies, and deep tendon reflexes. Moreover, a sural nerve biopsy in those patients reports a loss in the small myelinated fibers, with the preservation of large myelinated fibers, resulting in loss of deep sensation and pain [1]. The clinical presentation of our patient in her early second decade of life with impaired sensation to pain, her recurrent complaints of the multiple episodes of joint swellings and unreported fractures along with the reported normal mental status, muscle tone, and nerve conduction studies suggest the diagnosis of HSAN type 5. Minde et al. reported that symptoms manifested early in homozygous mutation carriers and were associated with more severe symptoms such as painless fractures and joint destruction [2]. Similarly, our patient was diagnosed with homozygous *NTRK1* gene mutation, which encodes the receptor tyrosine kinase for nerve growth factor. Mutations in the *NTRK1* gene are associated with congenital insensitivity to pain with anhidrosis [8]. Symptoms appeared early during childhood with multiple orthopedic complaints leading to further investigations and diagnosis [8]. Though our patient had mainly knee joint abnormalities dominating the clinical picture, wrists, foot, and ankle arthropathies have also been manifested but were neglected mainly due to lack of pain and the lack of massive swelling in comparison to the knee. Charcot arthropathy is a relatively rare, poorly understood condition known to result mainly because of diabetes [6]. Charcot arthropathy can result from repetitive microtrauma and autonomic dysfunction. It commonly affects the foot and ankle and is rarely observed in the knees [6]. Historically, neuropathic arthropathy has been treated conservatively or with arthrodesis, while arthroplasty of the Charcot joint has generally been avoided. However, when performed correctly and with the appropriate technique to the right patient, arthroplasty of the Charcot joint has been shown to significantly improve symptoms [3]. Moreover, arthroplasty has been shown to be superior to arthrodesis in cases of Charcot knees [3]. This is mainly because it restores normal knee alignment, provides better ability in bearing weight, and helps in early mobilization in the rehabilitation course with higher knee function scores [4]. Taking into consideration the rarity of Charcot knees, with only a few cases reported in the literature, and the highly variable presentation, Charcot knee diagnosis can be challenging, and a delay in the diagnosis can lead to advancement of joint destruction with further surgical complications [4,6]. However, in most cases of neuropathic arthropathy, a thorough clinical history and physical examination supported by X-rays showing signs of knee subluxation or dislocation along with bony destruction or heterotopic ossification is sufficient to make Charcot knee diagnosis without the need for advanced imaging modalities such as magnetic resonance imaging (MRI) [4]. While painless arthropathy has been described as the classical Charcot knee clinical presentation, knee pain in addition to instability and decreased range of motion have been considered to be the two major presenting complaints of patients suffering from Charcot knees [4]. However, our patient presented with instability and limping without reporting any pain [3,4]. To our knowledge, there are no guidelines addressing the management and approach of Charcot knees due to HSAN in adolescent patients. Therefore, further studies need to be conducted in order to establish a clear guideline for similar patients. Moreover, follow-up and monitoring for our patient are recommended to determine the long-term outcome of the approach.

## Conclusions

Patients with HSAN can develop several orthopedic complications due to the loss of deep pain sensation, which makes them more prone to multiple traumas and several minor fractures for which they may not seek medical attention. Complications can develop early in childhood and adolescence if the patient has homozygous mutations in the genes causing HSAN, as in the case of *NTRK1* gene mutation. Classical joint neuropathic arthropathy is painless and known to be associated with a secondary cause, for example, diabetes mellitus and neurosyphilis. However, Charcot knee joint is known to be associated with pain and discomfort. Therefore, in the absence of pain, possible secondary cause, and in the presence of HSAN, diagnosing Charcot joint may be delayed and can be very challenging. Charcot knee arthroplasty is becoming a viable option in patients with HSAN who develop Charcot knees and has been shown to be beneficial for the patient.
